# Real-world clinical utility of tumor whole-genome sequencing in solid cancers

**DOI:** 10.1038/s41591-026-04280-2

**Published:** 2026-03-20

**Authors:** Jeffrey van Putten, Petur Snaebjornsson, Linda J. W. Bosch, Roelof Koster, Paul Roepman, Joseph Usset, Mirjam C. Boelens, Tom van Wezel, Efraim H. Rosenberg, Serena Marchetti, Marieke Vollebergh, Doenja M. J. Lambregts, Lizet E. van der Kolk, Edwin Cuppen, Hilde H. Nienhuis, Kim Monkhorst

**Affiliations:** 1 Hartwig Medical Foundation, Amsterdam, The Netherlands; 2 Department of Pathology, Netherlands Cancer Institute, Amsterdam, The Netherlands; 3 Faculty of Medicine, University of Iceland, Reykjavik, Iceland; 4 Department of Pathology, Leiden University Medical Center, Leiden, The Netherlands; 5 Department of Medical Oncology, Netherlands Cancer Institute, Amsterdam, The Netherlands; 6 Department of Radiology, Netherlands Cancer Institute, Amsterdam, The Netherlands; 7 Department of Clinical Genetics, Netherlands Cancer Institute, Amsterdam, The Netherlands; 8 Center for Molecular Medicine, University Medical Center Utrecht, Utrecht, The Netherlands; 9 Department of Medical Oncology, University Medical Center Utrecht, Utrecht, The Netherlands

**Keywords:** Cancer genomics, Cancer of unknown primary

## Abstract

Molecular testing is essential in precision oncology. Whole-genome sequencing (WGS) provides a tumor-agnostic solution for detecting an increasingly complex range of DNA-based biomarkers. Here we present real-world data from 888 patients to demonstrate the clinical utility of routine, paired tumor-normal WGS diagnostics for solid cancers in a comprehensive cancer center. WGS succeeded in 89% of cases with a median turnaround time of 6 working days. Potentially actionable biomarkers were identified in 73% of patients, including biomarkers for reimbursed (27%) and experimental (63%) therapies. Within 1 year, 40% and 19% of patients, respectively, started biomarker-informed treatment, which was associated with a 31% longer median overall survival (*+*96 days) compared with patients not receiving such therapy. Among patients without prior systemic therapy, biomarker-informed treatment yielded significantly longer overall survival (median not reached) than non-biomarker-informed therapy (427 days) or no systemic therapy (214 days). In cancers of unknown primary (*n* = 123), WGS contributed to diagnostic solution or detected biomarker-driven reimbursed treatment options in 67%, with 68% starting tumor-type-specific therapy. Clinically relevant pathogenic germline variants were identified in 6.5% of patients. Overall, WGS-based diagnostics had clinical consequences for 41% of tested patients, providing a versatile tool for routine clinical practice in solid oncology.

## Abstract (web summary)

In a real-world cohort of 888 patients with solid cancers receiving whole-genome sequencing profiling, actionable biomarkers were identified in 73% of patients, and targeted therapies were associated with clinical benefit in patients without prior systemic therapy.

## Main

In recent decades, advances in molecular diagnostics have transformed cancer care by enabling precision oncology^1–3^. Traditional uniform treatment strategies have been replaced by approaches that integrate molecular biomarkers to indicate prognosis^4,5^, predict therapeutic response or resistance^6,7^ and anticipate toxicity^8,9^. These biomarkers now play a central role in clinical decision-making^3–5^, underscoring the need for diagnostic platforms that can deliver comprehensive, accurate and efficient testing.

Historically, sequential single-biomarker assays formed the cornerstone of molecular diagnostics, but this approach is limited by tissue availability, cost and turnaround time. High-throughput sequencing technologies have addressed many of these challenges^2,3,10^, and panel-based next-generation sequencing (NGS) is now recommended by international guidelines for many advanced cancers^11,12^. However, even broad NGS panels can fail to capture complex genomic alterations and are slow to incorporate new biomarkers due to regulatory and validation requirements.

Whole-genome sequencing (WGS) has emerged as a transformative alternative^13^. Unlike targeted NGS panels, WGS inherently captures the full spectrum of clinically relevant alterations, which includes complex structural variants^14^, single-exon deletions^15^, pathogenic germline variants (PGVs)^16^, homologous recombination deficiency (HRD)^17–20^ and emerging tumor-agnostic biomarkers within a single assay. Clinical validation studies have demonstrated high sensitivity and concordance with standard diagnostics^15,21^, and clinical utility has been shown across diverse patient populations, including children and young adults^22–24^, patients with rare or metastatic cancers^25^ and those with hematological malignancies^26^. Importantly, WGS has demonstrated added value for cancers of unknown primary (CUP)^27^, for which reimbursement has already been implemented in the Netherlands^28^.

Despite these advantages, real-world adoption of WGS remains heterogeneous. Large-scale national initiatives, such as the 100,000 Genomes Project in England^29,30^, have demonstrated the feasibility of WGS at scale. In the Netherlands, multidisciplinary research consortia have driven implementation and secured reimbursement for selected tumor types by defining the clinically required biomarkers that need to be tested in professional guidelines^31^, while leaving the decision on testing strategies to hospitals. This guideline-based approach has facilitated broader adoption of WGS, including reimbursement for non-small cell lung cancer (NSCLC)^32^, and facilitates further extension of indications as more tumor-agnostic biomarkers become part of standard care^33^. Yet widespread integration remains constrained by challenges^13,34^, including the requirement for high-quality input material that is not damaged by formalin-fixed paraffin-embedded procedures, computational complexity and higher costs compared with panel-based sequencing^10,24^, even though workflow adaptations can mitigate these challenges and decrease costs^14,26,29,34–36^. Furthermore, although the clinical utility of panel-based NGS has been increasingly demonstrated across cancer types^37–41^, the impact of WGS on patient-level outcomes has not yet been systematically evaluated.

An early implementation study at the Netherlands Cancer Institute (2018–2020) established the feasibility of WGS in 1,200 patients with solid tumors, reporting a 70% success rate, a median turnaround time of 11 working days and actionable findings in 71% of patients, with treatment changes in nearly a quarter^25,42^. Building on these results, the Netherlands Cancer Institute further improved and optimized pathology workflows in 2021^34^ and became the first hospital in the country to integrate WGS into routine oncology practice for patients with metastatic, rare or poor-prognosis cancers.

Here we report on the performance, feasibility and clinical utility of WGS after its integration into routine clinical care, as well as its association with survival outcomes over the first 2 years of implementation.

## Results

### Feasibility in routine cancer diagnostics

The investigated cohort comprised of 935 unique patients for whom WGS was requested between January 2021 and November 2022. The mean age at sequencing was 60.9 years, with 54% of patients being female. The most common cancer types were NSCLC (23%), CUP (16%) and soft tissue sarcoma (10%) (Supplementary Table 1a).

The suitability of tissue samples for WGS was based on cryosection analysis of fresh frozen biopsies or surgical specimens. A pathologist annotated viable tumor cells for manual microdissection to enrich the tumor cell percentage of all samples. If samples were too small for automated isolation with a robot (<6 mm), in-house DNA extraction was performed to improve sequencing feasibility for smaller samples^34^. As 90 samples lacked any tumor cells in the cryosection, 888 suitable tissue samples could be prepared for WGS.

**Fig. 1:**
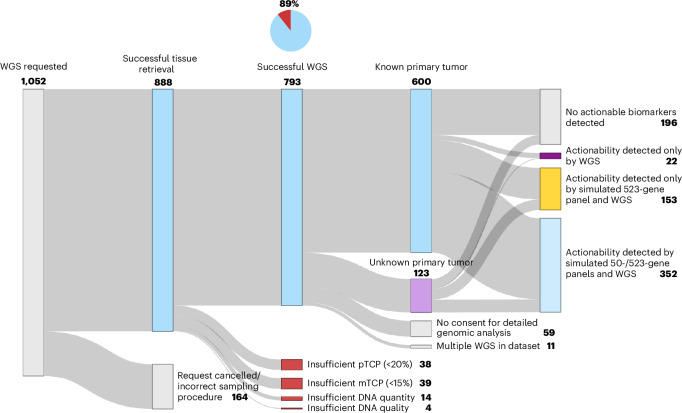
Feasibility of WGS in routine clinical practice and simulated actionable biomarker coverage by small and comprehensive sequencing panels. Starting from 1,052 total WGS requests, reasons for discontinuation and failure as well as diagnostics outcomes are indicated. Patients who did not have consent for patient-level genomic data analysis were included only for general patient characteristics. Panel coverage of a commonly used 50-gene panel and a 523-gene panel was simulated. Potential actionability includes standard-of-care and experimental biomarker-informed treatments. pTCP, pathologist-assessed tumor cell percentage. Source Data

**Extended Data Fig. 1:**
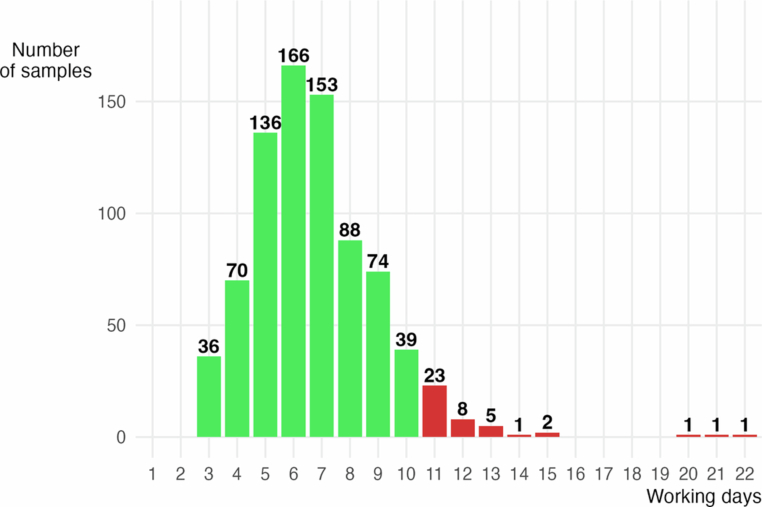
Turnaround time of WGS. Available timestamp data of 804 samples was retrieved from the external sequencing facility. Turnaround time is counted as time from material reception at the external laboratory to reporting to the clinic. Samples with a turnaround time of ten or fewer working days are indicated in green, and samples with turnaround times exceeding ten working days in red. Source Data

Successful diagnostic sequencing reports were generated for 89% (793 of 888) of samples (Fig. 1). Cytology samples achieved a lower success rate (57%; 20 of 35) than surgical specimens or biopsies (91%; 773 of 853) (Supplementary Table 1b,c). WGS performed on archived samples achieved comparable success rates (90%; 135 of 150). Notably, the median turnaround time was 6 working days (average, 6.7; range, 3–22) from sample reception at the sequencing facility until reporting (Extended Data Fig. 1).

Among 95 samples that failed to yield WGS reports, 40% (38 of 95) contained an insufficient pathologist-assessed tumor cell percentage (<20%) despite enhancement of tumor cellularity^34^. These samples were not processed for sequencing. Although WGS normally requires a minimum molecular tumor cell percentage (mTCP, based on sequencing results) of 20%, previous analyses indicated that mTCPs between 15% and 20% maintained a sensitivity of more than 95% without increased false positives. We followed institutional practice in our analyses by adopting this mTCP threshold of 15% while disclaiming in the patient report that biomarkers may have been missed when mTCP was between 15% and 20%. Forty-one percent (39 of 95) had an mTCP below 15% after sequencing and were thus considered failed. Finally, 15% (14 of 95) had insufficient DNA quantity (<50 ng), and 3% (4 of 95) had library preparation failures due to inadequate DNA quality.

### Clinical actionability and simulation of panel-based testing

Next, we investigated the presence of potentially clinically actionable biomarkers identified through WGS. We focused on biomarkers indicating positive or negative selection for available reimbursed or experimental treatments, as registered in the electronic health record (EHR) following molecular tumor board (MTB) discussion.

A total of 793 WGS reports were generated, but consent for patient-level analysis of EHR data was available for only 723 patients (Table 1 and Supplementary Table 1b). Eleven patients had multiple WGS analyses during their patient journey (Supplementary Table 2b).

**Table 1:** Patient characteristics and tumor types

**Known primary tumor** (*n* | %)	600	83.0
Age (mean; range)	60.4; 19.7–85.3	
Female sex (*n* | %)	322	53.7
Diagnosis after WGS	*n*	*%*
Anus	1	0.1
Benign tumor	1	0.1
Biliary tract	1	0.1
Bladder	10	1.4
Bone	4	0.6
Brain/CNS	5	0.7
Breast	70	9.7
Cervix	11	1.5
Colorectal	42	5.8
Esophagus/stomach	21	2.9
GIST	5	0.7
Head and neck	9	1.2
Kidney	8	1.1
Liver	1	0.1
Lung (carcinoid)	22	3.0
Lung (LCNEC)	5	0.7
Lung (NSCLC)	150	20.7
Lung (SCLC)	2	0.3
Melanoma	7	1.0
Mesothelioma	36	5.0
Myeloproliferative neoplasm	1	0.1
Ovary	27	3.7
Pancreas	3	0.4
Pancreas (NEC)	2	0.3
Pancreas (NET)	2	0.3
Penis	4	0.6
Prostate	32	4.4
Salivary gland	2	0.3
Skin	3	0.4
Skin adnex	1	0.1
Small intestine	2	0.3
Soft tissue	72	10.0
Teratoma^a^	1	0.1
Testis	2	0.3
Thymus	3	0.4
Thyroid	2	0.3
Urachus	1	0.1
Urothelial tract	14	1.9
Uterus/endometrium	13	1.8
Vagina	1	0.1
Vulva	1	0.1
**Cancers of unknown primary** (*n* | %)	123	17.0
Age (mean; range)	63.0; 24.7–87.1	
Female sex (*n* | %)	65	52.8
**Total**	723	100

Tumor-type distribution of 723 patients with a successful WGS report, of whom 600 had a known primary tumor type and 123 had a CUP. Bold text indicates the two diagnosis-type groups (known primary tumors and CUP). ^a^Teratoma subtyped as immature extra-ovarian teratoma. CNS, central nervous system; GIST, gastrointestinal stromal tumor; LCNEC, large cell neuro-endocrine carcinoma; NEC, neuro-endocrine carcinoma; NET, neuro-endocrine tumor.

**Extended Data Fig. 2:**
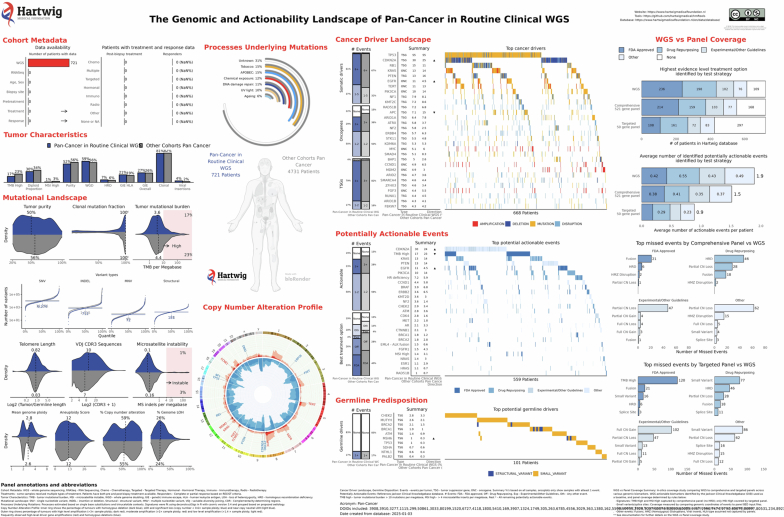
Pan-cancer ‘vignette’ visualising genomic differences between the real-world WGS cohort and patients in the Hartwig Medical database prior to January 2021. Data of 721 of 723 patients of the cohort were stored in the database, which were compared to 4,731 other patients with whole-genome data in the Hartwig Medical database. Panels include tumor characteristics, general markers of mutational landscape (leftmost panels), signatures and copy number alterations (center-left panels), cancer drivers, actionable biomarkers, and germline alterations (center-right panels), and in silico comparison between WGS and small (targeted) and comprehensive panels (rightmost panels).

**Extended Data Fig. 3:**
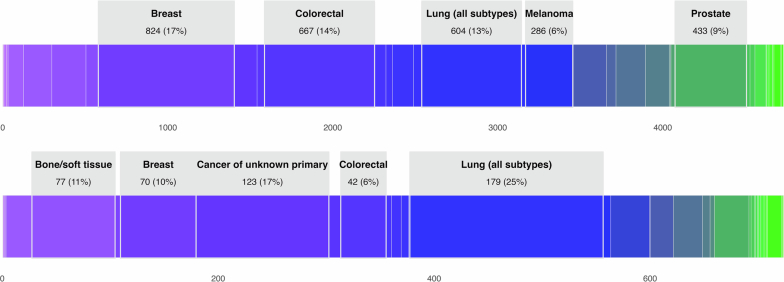
Tumor-type distribution of the Hartwig Medical database (top) as of January 2021, and the routine clinical WGS cohort described in this study (bottom). Top 5 cancer types in both cohorts are indicated with grey boxes containing numbers and percentages of the total number of patients in each. Cancer types are grouped as registered in the Hartwig Medical database (for example, Bone/soft tissue rather than as separate primary tumor types) to allow easier comparison with the Hartwig dataset. Source Data

**Extended Data Fig. 4:**
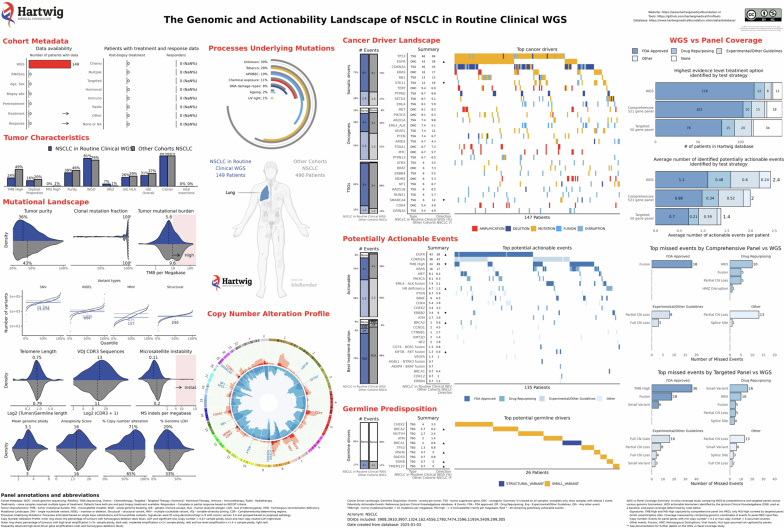
An NSCLC ‘vignette’ as an example of whole-genome visualisation for a specific cancer type. Data of 149 of 150 NSCLC patients in the cohort were stored in the Hartwig Medical database, and compared to 490 other NSCLC patients in the Hartwig Medical database as of January 2021. Panels include tumor characteristics, general markers of mutational landscape (leftmost panels), signatures and copy number alterations (center-left panels), cancer drivers, actionable biomarkers, and germline alterations (center-right panels), and in silico comparison between WGS and small (targeted) and comprehensive panels (rightmost panels).

Overall, we found that 73% (527 of 723) of patients harbored at least one potentially actionable biomarker. An overview of cancer drivers and actionable biomarkers can be found in the supplementary data (Supplementary Fig. 1 and Supplementary Table 2a,c–e) as well as in the comprehensive ‘cancer vignette’ visualization of the genomic landscapes of all patients in this study and the NSCLC subcohort. In these visualizations, genomic characteristics of the cohort described in this study are compared with data of ~5,000 patients present in the Hartwig Medical database (Extended Data Figs. 2–4), demonstrating that patients in the current study are representative from a genomics perspective.

We simulated the coverage of commonly used 50-gene (small) and 523-gene (comprehensive) NGS panels within our WGS dataset and show that these test approaches would detect actionable biomarkers in only 49% (352 of 723) and 70% (505 of 723) of patients, respectively (Fig. 1 and Supplementary Table 3a–c). Furthermore, in 8% (40 of 505) of patients for which actionable biomarkers could be detected using a comprehensive panel, WGS identified additional actionable biomarkers. In 3% of patients (22 of 723), no potentially actionable biomarkers would have been identified using panel-based NGS, yet WGS revealed clinically relevant alterations; these were predominantly gene fusions (*n* = 11) and HRD in the absence of pathogenic mutations in known homologous recombination pathway genes (*n* = 7). Collectively, these findings suggest that about one in ten patients may benefit from the broader actionability detection of WGS compared with comprehensive panel-based screening.

We note that patients with CUP represent a unique subcohort. As WGS serves a critical role in establishing the initial cancer diagnosis, delivery of non-biomarker-informed systemic therapy due to demystification of the tumor type is as relevant as finding an actionable biomarker. We therefore analyzed patients with known primary tumor diagnoses (*n* = 600) and patients with CUP (*n* = 123) separately.

### Actionability in patients with known primary tumors

**Fig. 2:**
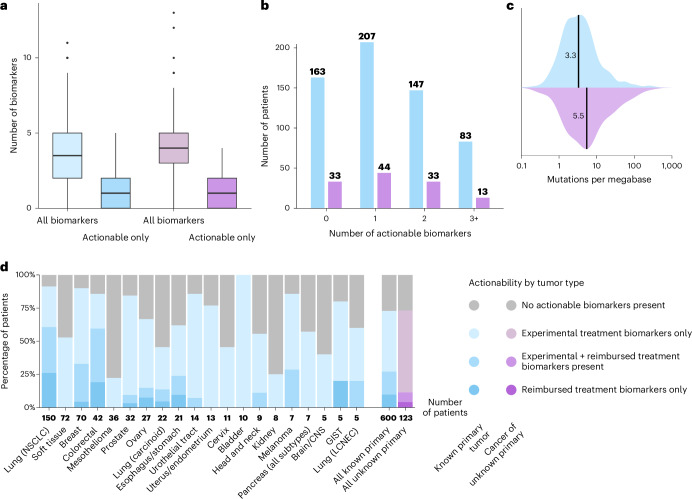
Overview of actionability. Data are shown separately for patients with known primary tumors (blue, *n* = 600) and CUP (purple, *n* = 123). **a**, Number of biomarkers and actionable biomarkers reported per patient. Boxes indicate the interquartile range (IQR), center lines the median and whiskers the extreme values within 1.5 × IQR. Outliers are represented by dots. **b**, Number of patients with zero, one, two or three or more actionable biomarkers. **c**, TMB distribution in mutations per megabase. The line indicates the median. **d**, Fractions of patients harboring actionable biomarkers per tumor type (with at least five representations in the cohort). Bars are proportioned to 100%. Source Data

Among 600 patients with known primary tumors, 73% (437 of 600) presented one or more clinically actionable biomarkers, averaging 1.3 actionable biomarkers per patient (median, 1; range, 0–5) (Fig. 2a,b). The median tumor mutational burden (TMB) was 3.3 mutations per megabase (Fig. 2c). Notably, 27% (162 of 600) of tumors exhibited biomarkers linked to reimbursed treatments, most commonly *EGFR* mutations (70 of 600). Sixty-three percent (379 of 600) of patients had biomarkers enabling experimental treatments, mostly based on a high TMB (≥10 mutations per megabase; 147 of 379) (Fig. 2d, Supplementary Fig. 1 and Supplementary Table 2c–e).

An average of 1.8 (range, 0–10) systemic treatments were given before WGS. Twenty-six percent (158 of 600) of patients had no prior systemic treatments, and 19% (114 of 600) had received a biomarker-informed treatment (Supplementary Table 2a).

### Biomarker-informed treatment in patients with known primary tumors

Next, we evaluated patient allocation to biomarker-informed treatments. We excluded 12 patients who had died or were no longer in care when WGS results were reported. The average follow-up was 10.1 months (median, 8.8; range, 0.0–28.7).

Twenty-seven percent (159 of 588) of patients had a biomarker for a reimbursed treatment, and 40% (63 of 159) of them did start such treatment. Notably, 21 of these were patients with NSCLC whose *EGFR-, ALK-, MET-, RET-* or *ROS1-*targeted tyrosine kinase inhibitor treatment was changed after WGS identified biomarkers suggestive of resistance.

A common reason not to start a reimbursed biomarker-informed treatment was that detected biomarkers contained no new actionable insights over previously initiated treatments (21%; 34 of 159). Additionally, three patients were enrolled in a clinical trial based on biomarkers for which a reimbursed treatment was also available. Another major reason not to pursue biomarker-informed treatment was related to ongoing response or complete remission after previous treatments, including surgery or radiotherapy (18%; 28 of 159). This number could thus change with longer follow-up. Finally, five patients did not start a reimbursed biomarker-informed treatment due to a poor performance status (Supplementary Table 2a).

Sixty-three percent (373 of 588) of patients had actionable biomarkers enabling experimental biomarker-informed treatments, with 19% (71 of 373) starting with this treatment. Sixteen percent (58 of 297) were allocated to a clinical trial, and 3% received biomarker-informed treatment through either a compassionate use program (8 of 373) or an early access protocol (5 of 373).

### CUP tissue-of-origin analysis and actionability

**Fig. 3:**
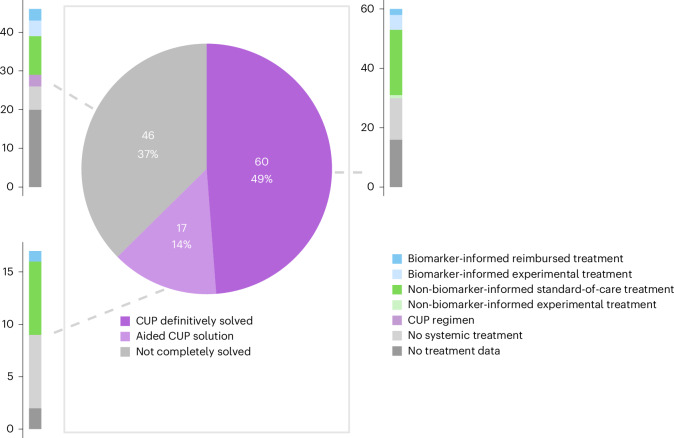
WGS-based resolution of CUP and subsequent systemic treatments. Treatment decision outcomes and frequencies and types of treatments administered following WGS are shown for all CUP patients analyzed (*n* = 123). For definitively solved CUP cases and those where WGS aided diagnostic resolution, biomarker-informed and non-biomarker-informed treatments represent tumor-type-specific therapies. In cases that were not fully solved, biomarker-informed and non-biomarker-informed treatments reflect treatments guided by tumor-type indications derived from WGS, even when some diagnostic uncertainty remained. Source Data

The outcomes of the WGS-based tissue-of-origin prediction algorithm (Cancer of Unknown Primary Prediction Algorithm (CUPPA)) are routinely reported for all patients^27^. We found that for 49% (60 of 123) of CUP cases, a tissue of origin could be predicted confidently (confidence score ≥0.80). In an additional 14% (17 of 123), a conclusive diagnosis was also achieved by combining findings with lower confidence scores with prior clinicopathological findings, including from radiology, nuclear medicine or endoscopy (Fig. 3). Collectively, WGS led to a definitive tumor-type diagnosis for 63% of CUP patients (77 of 123; Supplementary Table 4a,b).

For 73% (90 of 123) of CUP patients, at least one potentially actionable biomarker was identified (Fig. 2a,b). After demystification of the tissue of origin, 11% (14 of 123) had biomarkers linked to reimbursed treatments, and 69% (85 of 123) had biomarkers linked to an experimental biomarker-informed treatment. Although these rates are similar to those in previous literature^27^, we noticed that definitively solved CUPs harbor fewer standard-of-care biomarkers than cancers that already had a clear diagnosis. For NSCLC patients with a previous CUP history, only 5% of cases (1 of 23) had an actionable biomarker, whereas this was 61% (91 of 150) for the directly diagnosed NSCLC patients. This raises the question of whether the lack of standard-of-care actionable pathway is related to diagnostic uncertainty in general or defines a separate tumor-type subgroup.

The median TMB in the CUP cohort was 5.5 mutations per megabase (Fig. 2c,d and Supplementary Table 2a) and slightly higher than tumors with known primary origin.

### Treatments in patients with CUP

Forty-seven percent (58 of 123) of CUP patients received a systemic treatment after WGS: 49% (38 of 77) following solved cases and 43% (20 of 46) following unsolved cases. However, more than one-third of CUP analyses (49 of 123) were done in the context of a second opinion and care after WGS diagnostics was mostly given in the referring hospital, with clinical follow-up not accessible for this study due to privacy regulations. When excluding 38 patients whose systemic treatment information was entirely unknown, we found that 64% (38 of 59) of solved cases received systemic treatment and 76% (20 of 26) of unsolved CUPs. Overall, 68% (58 of 85) of cases with known treatment data received a systemic treatment after WGS.

Most CUP patients were allocated to non-biomarker-informed standard-of-care treatments based on new WGS-derived diagnostic insights (46%; 39 of 85). A smaller proportion received biomarker-informed reimbursed treatments (7%; 6 of 85) or were enrolled in clinical trials for biomarker-informed experimental treatments (11%; 9 of 85). One patient was assigned to a non-biomarker-informed clinical trial, and three patients whose diagnoses remained unresolved received empirical CUP regimens (Fig. 3 and Supplementary Table 4a).

Although tissue-of-origin prediction was not a primary objective for the 600 patients with known primary tumors, the CUPPA analysis is part of the standardized WGS data analysis workflow for all patients. Interestingly, the algorithm provided added diagnostic value for 3% (20 of 600) of patients with established diagnoses, mainly resulting in improvements of soft tissue sarcoma subtyping. This had direct treatment consequences for 13 of 20 patients. Furthermore, for an additional six patients (1%; 6 of 600), a complete revision of an established diagnosis was made, with immediate treatment implications for one (Supplementary Table 4c).

### Detection of PGVs

Finally, we analyzed the identification of clinically relevant PGVs that can be found because the WGS tests includes a normal blood sample as control for somatic variant detection. Consent for germline analysis was available for 96% of patients (692 of 723). Following updated national guidelines, PGVs were reported as present in the tumor without explicitly stating germline status^43^, which is why we performed this analysis in a post hoc setting. When a variant was identified as a potentially relevant PGV during MTB discussion, patients were referred for genetic counseling and germline analysis, leveraging pre-existing WGS data.

**Extended Data Fig. 5:**
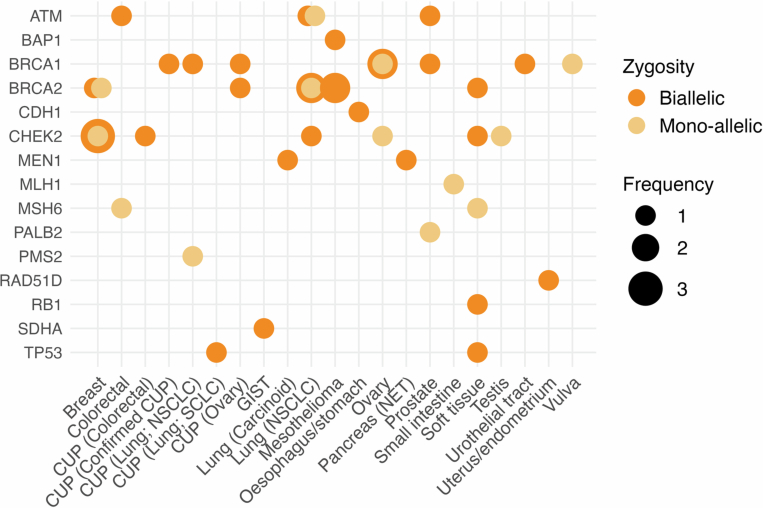
Detected pathogenic germline variants. Pathogenic germline variants are organised by cancer type. Orb sizes indicate how often variants were found in the cohort with appropriate consent for germline analysis (n = 692). Colors indicate differences in zygosity in individual tumors. CUP, cancer of unknown primary; NSCLC, non-small cell lung cancer; SCLC, small cell lung cancer; GIST, gastrointestinal stromal tumor; NET, neuro-endocrine tumor. Source Data

Overall, PGVs could be identified in 6.5% (45 of 692) of patients. About half of these variants had not been previously detected through routine diagnostics (23 of 45). Most showed a second somatic hit in the tumor (33 of 45). However, 12 did not show a second hit, and in one case, a confirmed pathogenic *BRCA2* germline variant was identified where the somatic hit had been lost in the tumor. No patient had more than one relevant PGV (Extended Data Fig. 5 and Supplementary Table 5).

### Overall clinical utility

To evaluate the overall utility of WGS in providing clinically valuable insights, we synthesized relevant results at a patient level: having a biomarker indicating reimbursed treatment, a WGS-based solution or improvement of a diagnosis, a clinically relevant PGV or a combination of these (Supplementary Table 6).

**Fig. 4:**
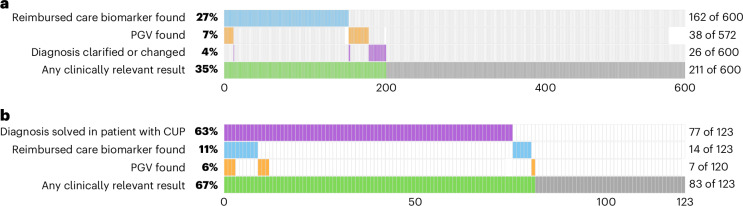
Combined utility of WGS per patient. Utility is synthesized (green) per investigated parameter (blue, reimbursed care biomarkers; orange, PGVs; purple, solved or clarified diagnosis). **a**,**b**, Six hundred patients with known primary tumor diagnoses (**a**) and 123 patients with CUP (**b**) are presented separately. Missing cells in the rows presenting PGV detection indicate patients who had indicated they did not want to be informed about potentially inherited variants. Source Data

WGS provided clinically relevant results for 35% (211 of 600) with known primary tumor diagnoses (Fig. 4a). For patients with CUP, this amounted to 67% (83 of 123) (Fig. 4b). Overall, clinically valuable insights were obtained for 41% of all patients (294 of 723) who received WGS-based molecular testing.

### Improvement of OS

**Fig. 5:**
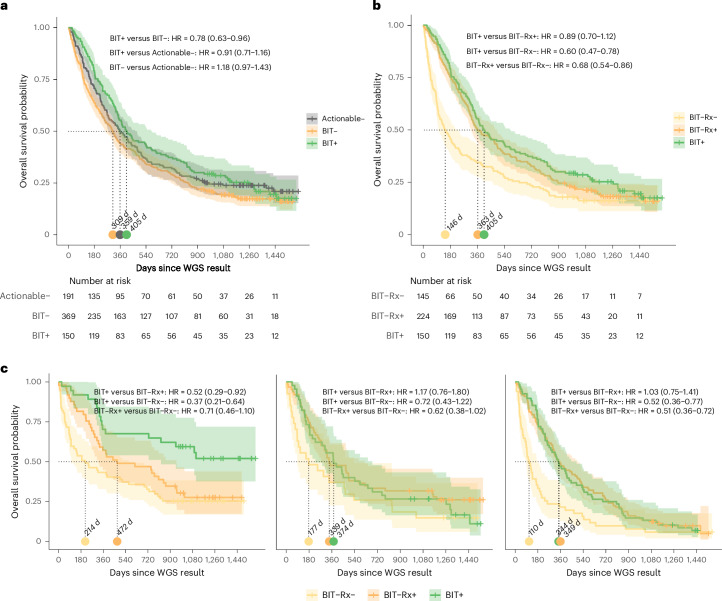
OS after WGS. **a**, Kaplan–Meier curves comparing patients without potentially actionable biomarkers (Actionable−), patients with ≥1 potentially actionable biomarker who did not receive a biomarker-informed treatment after WGS (BIT−) and patients with ≥1 potentially actionable biomarker who did receive biomarker-informed therapy (BIT+). **b**, Within the actionable cohort, patients not receiving biomarker-informed therapy are split into those receiving only non-biomarker-informed systemic therapy (BIT−Rx+) and those without any post-WGS systemic therapy (BIT−Rx−), shown alongside patients who received biomarker-informed treatment (BIT+). **c**, Among patients with ≥1 potentially actionable biomarker, OS is stratified by the number of prior systemic therapy lines at the time of WGS: 0 (left), 1 (center) or ≥2 (right). Dotted lines indicate the median survival and are annotated with the corresponding number of days. HRs are reported with 95% CIs in parentheses. BIT, biomarker-informed treatment; Rx, non-biomarker-informed systemic treatment. Source Data

Finally, we investigated overall survival (OS) after WGS in patients with survival data (*n* = 719) (Supplementary Table 7). OS was improved in patients with at least one potentially actionable biomarker who received biomarker-informed therapy after WGS compared with those harboring an actionable alteration who did not receive such treatment (hazard ratio (HR) = 0.78; 95% confidence interval (CI) 0.63–0.96). The median increased from 309 to 405 days (*+*31%), favoring the treated group. Median OS of patients without actionable biomarkers did not differ significantly from either actionable-biomarker subgroup (Fig. 5a).

In a subanalysis that split the group not receiving biomarker-informed therapy into patients receiving no treatment at all or receiving systemic therapies (Fig. 5b), biomarker-informed therapy remained superior to no therapy (HR = 0.60; 95% CI 0.47–0.78), with an almost threefold longer median OS (146 versus 405 days), whereas the difference versus non-biomarker-informed systemic therapy was smaller (*+*11%) and no longer significant.

When stratifying by the number of prior systemic treatment lines at the time of WGS (Fig. 5c), the OS benefit of biomarker-informed therapy was concentrated in patients without prior systemic therapy. These patients (median OS not reached after four years) outperformed those receiving only non-biomarker-informed systemic therapy (median OS 427 days; HR = 0.52; 95% CI 0.29–0.92) as well as those receiving no systemic therapy (median OS 214 days; HR = 0.37; 95% CI 0.21–0.64) despite having actionable biomarkers. By contrast, among patients with ≥1 prior systemic line, biomarker-informed therapy did not confer an OS advantage over non-biomarker-informed therapy.

These patterns were consistent irrespective of whether pre-WGS treatments had already included biomarker-informed therapy (Supplementary Fig. 2a–c). We also observed no significant OS differences between patients with CUP and known primary tumors (Supplementary Fig. 2d).

As these analyses represent real-world, non-randomized data, the prognostic and predictive effects of biomarkers for survival and treatment effects cannot be discriminated, and groups were not adjusted for baseline clinical condition (for example, performance status).

## Discussion

This real-world study shows that routine WGS for patients with solid cancers is feasible at scale and delivers substantial clinical impact. Compared with our earlier implementation study, test success increased from 70% to 89%, and median turnaround times decreased from 11 to 6 working days, now comparable to panel-based NGS^25,42^. These improvements were enabled through workflow optimizations in biopsy retrieval and tissue assessment^34^ and demonstrate that WGS can meet diagnostic requirements in routine oncology practice.

WGS provided clinically relevant results in 41% of patients, including 35% of patients with known primary tumors and 67% of patients with CUP, and enabled biomarker-informed treatments that were associated with improved OS. Furthermore, our analysis exemplifies how routinely generated genomic and clinical data can be reused to support learning and improvement of the healthcare system.

In 73% of patients, at least one potentially actionable biomarker was identified, which is consistent with previous literature^23,25,44,45^. Simulation of broad panel-based NGS showed that in about one in ten patients, one or more potentially actionable biomarkers would have been missed. Furthermore, 39% of patients with a reimbursed biomarker-informed treatment option and 19% of patients with experimental options detected by WGS actually received biomarker-informed therapy, similar to figures presented in other studies^25,38,46^. Late-stage use of WGS, prior molecular diagnostics, poor performance status and referral to other hospitals were the most likely reasons why no biomarker-informed therapy was given. Also, patients referred elsewhere may still have benefited from WGS-derived actionability findings. Although tumor genomes evolve over time and therapeutic pressure contributes to changes in mutational landscapes and clonal expansions, previous work has shown that main cancer drivers and actionability landscapes remain largely consistent, including after exposure to biomarker-directed and non-biomarker-directed treatments^47,48^. Because follow-up outside our center was unavailable and external treatment data were unavailable for ~20% of patients, actual treatment uptake may have been either under- or overestimated, underscoring the need for legal frameworks that support efficient and standardized multicenter data integration.

These analyses were performed in a tertiary referral center where WGS is often requested after other diagnostics or treatments have been exhausted, supported by our finding that an average of 1.8 systemic therapies had been administered before WGS was initiated in patients with known primary tumors. This contrasts with pediatric oncology, where extensive molecular testing including WGS is increasingly used as standard of care in early diagnostics^49^. Our real-world cohort of adult solid cancers reflects a case mix influenced by evolving referral patterns, reimbursement policies (for example, for CUP) and clinicians’ preference for WGS over panel-based sequencing when both are available, which may limit the generalizability of clinical value presented here. Furthermore, we defined clinical actionability dynamically, using MTB-based interpretation guided by available reimbursement and local biomarker-informed trial availability, rather than static frameworks such as Food and Drug Administration approval or the European Society for Medical Oncology Scale for Clinical Actionability of molecular Targets (ESCAT)^50^. Although this also limits generalizability, real-world actionability ultimately depends heavily on local drug accessibility, and international or time-independent definitions would likely overestimate the clinical utility of WGS results. Expert interpretation (supported by knowledgebase annotation) as the primary measure for actionability is potentially more subjective but in our opinion comes closest to real-world clinical utility.

Our survival analyses suggest that earlier use of WGS may matter. Previous work suggests that applying broad molecular diagnostics in early disease stages is clinically beneficial only for rare cancers^41,51,52^, as these patients depend largely on experimental, often off-label treatments^52^. However, in our mixed cohort of common and rare cancers, patients with actionable biomarkers who received biomarker-informed therapy after WGS had a 31% longer median OS than those who did not, which is consistent with findings from off-label biomarker-directed treatment studies such as the Drug Rediscovery Protocol study^53–55^. Importantly, the OS advantage of biomarker-informed therapy was primarily driven by patients without prior systemic therapy, whereas no advantage was seen after one or more treatment lines before WGS. These findings may support positioning WGS-based diagnostics early in the diagnostic pathway, rather than as a last-resort test. A note of caution is that these real-world, non-randomized data represent an aggregation of survival benefits achieved by the given treatments following WGS diagnostics, not of the diagnostic test itself. Considering that many biomarker-informed therapies are approved based on surrogate endpoints rather than improved OS^56^, judging the clinical utility of WGS by OS may be too narrow a framing.

Indeed, several clinically relevant parameters are not fully captured by survival outcomes. For patients with CUP, the largest impact is on ending the diagnostic odyssey, resolving or refining the diagnosis in more than two-thirds of patients and often enabling tumor-type-specific treatments, which is similar to other studies^27^. We noted that many patients returned to their original hospital upon completion of CUP diagnostics, limiting available clinical follow-up data, which may lead to underestimation of treatment figures for CUP patients. WGS-based tissue-of-origin prediction also resulted in revision of established diagnoses (1%) and improved subtyping (3%) in patients with a known primary tumor. Diagnostic revision following molecular testing has been reported in up to 10% of sarcoma patients^27,57,58^, but such figures are not yet available for other cancers. The potential impact of diagnostic revisions includes the evasion of adverse effects for patients and costs for society of ineffective treatments, which are not reflected in OS statistics. Furthermore, WGS facilitated identification of clinically relevant PGVs in 6.5% of patients^43^, around half of which had not been detected previously by routine genetic diagnostics. To comply with professional guidelines, germline status of variants is not disclosed in molecular diagnostic reports. However after clinical genetics counseling, clinically validated tumor-normal WGS data could be used to confirm suspected PGVs, saving both costs and time.

Despite the benefits described here and a range of European national initiatives driving WGS-based diagnostics in both clinical genetics and oncology, costs remain a key barrier to broad adoption of WGS. Although earlier microcosting studies have indicated that WGS is still more costly^36,59^, sequencing costs are decreasing with the emergence of new sequencing platforms including Illumina NovaSeqX, Ultima Genomics Solaris and Roche Axelios. Moreover, direct test costs do not fully capture downstream savings, such as reduced costs in the clinical genetics follow-up. In addition, WGS generates a rich data resource for the discovery of previously unknown biomarkers for patient stratification^60,61^ or non-responders^6^, which can further optimize the use of existing drugs and decrease total treatment costs for society. A learning healthcare system in which complete genomic data are linked with clinical data on treatments, outcomes and quality of life may thus be essential for both innovation in and sustainability of cancer care.

Realizing this potential will require timely integration of WGS into care pathways, centralized (data) infrastructure, harmonized reimbursement and guideline recommendations, clinical integration of MTB-guided decision-making and prospective collection of structured treatment and outcome data. Finally, national and international data platforms should be established to aggregate WGS results with clinical metadata, not only to record these data but also to make the data available for clinical and fundamental researchers.

In conclusion, real-world evidence from routine oncology now confirms the clinical value of WGS that has been observed in study settings. WGS improves care for current patients and generates unique data needed to optimize care for future patients, underscoring its role as a cornerstone of precision oncology. Broader implementation will depend on aligned policies and infrastructure that enable learning healthcare systems in which genomic and clinical data are continuously linked and reused so that patients can fully benefit from this transformative technology.

## Methods

### Compliance with ethical regulations and institutional review board approval

The study outline was reviewed and approved by the Institutional Review Board of the Netherlands Cancer Institute (NKI; IRBd22-294) in November 2022. The authors confirm that the research presented here complies with all relevant ethical regulations for scientific research in humans. All retrospective medical data and biospecimen studies at the NKI have been executed pursuant to Dutch legislation and international standards. Before 25 May 2018, national legislation on data protection applied, as well as the International Guideline on Good Clinical Practice. From 25 May 2019, studies also adhere to the General Data Protection Regulation. Within this framework, patients are informed and have always had the opportunity to object or actively consent to the (continued) use of their personal data and biospecimens in research. Hence, the procedures comply with both national and international legislative and ethical standards.

### Patient consent

Patient-level analyses of clinical and genomic findings were performed only for patients who had given explicit consent for reuse of their pseudonymized data for research purposes (opt-in, ‘consent at the gate’), within the legal framework described above.

Among the 1,052 clinical WGS requests identified in the EHR, 989 requests were from patients with explicit consent and were thus eligible for patient-level analysis. Data from the 63 requests for which such explicit consent was unavailable were used only in aggregated form (for example, in descriptive statistics of age, sex, diagnosis and WGS workflow outcomes for the full cohort), and no individual-level data from these patients are reported. This explicit consent was also required to store genomic data following WGS in the Hartwig Medical database and to request survival data from the national Personal Records Database in case no date of death was known within the NKI.

### Study design, study setting and tissue sample retrieval

This study is a single-center retrospective observational study in patients who received WGS as part of routine clinical diagnostics at the NKI and is reported in accordance with the Strengthening the Reporting of Observational Studies in Epidemiology guidelines.

WGS procedures were requested directly by clinicians through a molecular diagnostics request form that is integrated into the EHR, HiX (Chipsoft). There were no predefined criteria for clinicians for requesting WGS other than an indication for broad molecular diagnostics, and as such WGS could be performed at any point in the diagnostic process, from early stages of diagnostics to a last-resort setting.

A list of all pathology reports linked to a diagnostic WGS request between January 2021 and November 2022 was extracted from the hospital systems. After manual curation to remove duplicate and non-eligible entries, a total of 1,052 unique clinical WGS requests were retained. Following these requests, 978 tissue sampling procedures were performed in 935 unique patients. Some patients had multiple tissue samples because WGS was requested on multiple occasions or because a previous attempt had failed. In each case, two to four samples were taken during a biopsy procedure, and frozen samples were prioritized by the pathologist based on suitability for WGS or formalin-fixed paraffin-embedded-based diagnostics. Eight hundred eighty-eight tissue samples were suitable for assessment of tumor cellularity. A frozen section of 5 μm thickness of each sample was microscopically assessed and enhanced through manual macrodissection to ensure optimized tumor cell percentages, with 20% being the minimum required to attempt WGS. Tissue samples that were too small for automated bead-based DNA isolation with a robot (<6 mm) were eligible for in-house manual DNA extraction using a column-based method. A minimum DNA yield of 50 ng was required for WGS library preparation. The full workflow including required materials and timeframes is detailed elsewhere^34^. The pathology department of the NKI is accredited by the Dutch Accreditation Council with ISO 15189:2012.

Blood samples were drawn as part of standard clinical laboratory procedures. After local processing, tissue samples and blood samples were transported in most cases within 24 hours after sampling to the external laboratory facility of Hartwig Medical Foundation (Amsterdam, The Netherlands; henceforth referred to as Hartwig).

### Sequencing process and reporting

WGS was performed at the laboratory of Hartwig on Illumina platforms with a sequencing depth of 90× for tumor DNA and 30× for normal DNA derived from the blood sample.

Analysis of sequencing data was performed with Hartwig’s open source, in-house bioinformatics pipeline (available through https://github.com/hartwigmedical). Hartwig operates under ISO 17025:2017 accreditation for laboratory procedures and under ISO 27001:2022 for data security. The bioinformatics pipeline was continuously kept up to date using annotated information from the genomic libraries of the JAX Clinical Knowledgebase (The Jackson Laboratory) and the iClusion clinical trial database. As such, treatment evidence related to reported variants and biomarkers could change over time. The OncoAct report of genomic findings contains all ±500 oncogenes and tumor suppressor genes relevant for diagnostics and clinical decision-making. All tumor samples were analyzed for gene mutations in exonic regions and three base pairs at either side into intronic regions. Detection of mutational signatures included exonic, intronic and intergenic regions (specifications available via https://www.oncoact.nl/specsheetOncoActWGS).

For germline analysis, single nucleotide variants and indels with a second hit in the tumor were reported. After updated national guidelines in September 2021, mono-allelic variants were also reported.

The detailed reports of genomic and germline findings and matched implications for biomarker-informed systemic therapies were shared with the department of pathology via a secured online platform. Copies of the files were stored in the EHR and in the pathology laboratory system.

### CUP diagnostics

Before WGS, all clinical information (various imaging, endoscopic procedures, serum tumor markers, clinical presentation, medical history) and histopathology findings (including immunohistochemistry profiles) were integrated to verify the diagnosis of CUP and determine clinicopathological differential diagnoses. Cases included presumed carcinomas, melanomas and sarcomas. All cases fulfilled the minimum criteria for the diagnosis of CUP according to European Society for Medical Oncology guidelines^62,63^.

High-confidence prediction by the CUPPA algorithm using WGS data was defined as ≥0.80 and low-confidence prediction as <0.80, as described elsewhere^27^. After WGS, all available diagnostic information was integrated, and it was determined whether the prediction corresponded with the clinicopathological differential diagnosis before WGS analysis or whether the prediction did not fit the clinical presentation of the patient or did not provide information aiding in the diagnosis of the primary tumor.

### Data extraction

Clinical data were extracted between April and August 2023. Data including patient characteristics, medical history, treatments before WGS and clinical actionability during the follow-up period, as well as clinically interpreted genomic data, were extracted from the pathology file and clinical file of the EHR. After receiving the detailed genomic reports, clinical molecular scientists at the NKI interpreted the variants and biomarkers of potential clinical relevance in the context of the patient. Clinically relevant genomic findings were registered in the pathology section or the medical history in the EHR after discussion in an MTB.

Sex was recorded as registered sex (male/female) in the EHR and summarized descriptively. No sex-stratified analyses were planned, as the retrospective, observational study was not designed or powered to detect sex-specific differences.

Data relating to tumor cell percentages were extracted from the pathology laboratory system. For sample logistics, the WGS request date was defined as *t* = 0 for each sample. Samples that had been retrieved and archived before the WGS request were represented as having negative time values. In analyses related to clinical actionability, *t* = 0 was defined as the WGS reporting date. Patients who had died or continued care in another hospital before reporting could therefore had a negative follow-up time.

Clinical actionability was defined as the presence of a biomarker with a corresponding systemic treatment option available either through standard-of-care reimbursement or via enrollment in an active clinical trial at the time of reporting and included cases of positive or negative selection for treatment (for example, administration or avoidance of *EGFR* inhibitors in *RAS/RAF*-mutated or wildtype left-sided metastatic colorectal cancer). Actionability was determined retrospectively by reviewing entries from MTB discussions, clinical scientists and clinicians in the EHR. As more treatments became available, clinical trials opened or closed and actionability definitions changed over time (for example, the definition of a high TMB changed from ≥10.0 to ≥16.0, after TML ≥ 140 had initially been used), the clinical actionability status of specific biomarkers was dynamic and could differ depending on the time of reporting.

Treatments were considered administered if initiation was explicitly documented in the patient file. Biomarker-informed treatments included both targeted therapies (for example, tyrosine kinase inhibitors directed at specific mutations) and other systemic therapies guided by the presence or absence of genomic biomarkers.

Conclusions of MTB discussions as registered in the EHR were analyzed to assess diagnostic resolution in CUP cases. Treatment data after CUP analysis were extracted from the clinical file of the EHR. Here WGS-informed treatments comprised both biomarker-informed therapies and non-biomarker-informed, tumor-type-specific treatments enabled by WGS-based diagnostics.

### Panel coverage simulation

To assess which clinically relevant variants would have been captured by commonly used NGS panels, we performed an in silico simulation of panel coverage. We used BED files representing two panels: a small 50-gene panel (AmpliSeq Cancer Hotspot Panel v2 (CHPv2); Thermo Fisher Scientific) and a comprehensive 523-gene panel (TruSight Oncology 500 (TSO500); Illumina). Variant positions were overlapped with the panel regions using BEDtools.

Events were considered covered if they fell within the target regions (for small variants) or if the gene was listed for copy number variant detection. Structural variants, gene fusions, and mutational signatures were assumed to be missed. HRD was considered missed by both panels unless a specific substrate event was explicitly covered, microsatellite instability was considered covered by both panels, and TMB-high was only considered covered by TSO500. Additional RNA-based or HRD add-ons for TSO500 were not included in the comparison. Classification logic was based on rules also used in the ‘vignette’ visualizations of the Hartwig Medical database (documentation accessible via https://www.hartwigmedicalfoundation.nl/wp-content/uploads/2024/07/vignettes_documentation.docx).

Using this approach, we classified 2,757 WGS-reported biomarkers from 723 patients and calculated coverage per panel, categorized as being linked to reimbursed biomarker-informed treatments, experimental biomarker-informed treatments and non-actionable biomarkers. Results were summarized per variant type, gene and diagnostic group.

### Analysis of germline variants

For the analysis of PGVs, only data available within our hospital was used and analyzed retrospectively. The data reflect a change in the reporting pipeline. For WGS performed in 2021, only biallelic germline variants were annotated in the report, regardless of cancer type. Later, as of September 2021, after updated national guidelines with a predefined list of PGVs relevant for specific cancer types^43^, reporting also included mono-allelic variants relating to carriership. Germline copy number variants and structural variants were not included in our analysis.

Patients who received WGS in 2021 were given the choice to be informed about the germline status of variants present in the tumor; for 31 patients who indicated they wished not to be informed about potentially inherited variants, germline findings were not considered for our analyses. As of 2022, the procedure was changed to follow the updated national guidelines, with patients now being informed before undergoing WGS that the test could indicate hereditary cancer predisposition. If a gene of potential relevance was found and reported, patients could be referred to a clinical geneticist for further analysis to confirm inherited predisposition. As such, EHR data relating to potentially relevant germline findings were available for 692 of 723 patients.

### Statistics and reproducibility

No statistical method was used to predetermine sample size. For descriptive analyses of tissue sample characteristics and WGS success rate, no data were excluded from the cohort as defined above. Patient-level analyses of clinical utility were restricted to patients who had provided informed consent, as described under ‘Patient consent’. The study was not randomized, and investigators were not blinded to group allocation during data collection or analysis, reflecting the retrospective, observational study design.

Data were organized and analyzed in Microsoft Excel and in R (version 4.4.2) using RStudio (version 2024.09.1 + 394; Posit PBC). Descriptive statistics were used to analyze patient and tissue sample characteristics, WGS success rate and clinical utility parameters. Categorical variables are reported as counts and percentages and continuous variables as means or medians with ranges, as appropriate. Data visualizations were generated using the following R packages: networkD3 (v0.4), htmlwidgets (v1.6.4), extrafont (v0.20), readxl (v1.4.5), ggplot2 (v3.5.2), tidyr (v1.3.1), dplyr (v1.1.4), readr (v2.1.5), scales (v1.3.0), stringr (v1.5.1), cowplot (v1.1.3), survival (v.3.7-0), survminer (v.0.5.0) and patchwork (v1.3.0).

OS was defined as the time from WGS reporting (*t* = 0) to death from any cause, with censoring at the date of registry check for living patients. OS data were obtained from the EHR or, if unavailable, requested from the national Personal Records Database. Patients who had died before the time of WGS reporting (*n* = 12) were excluded from OS analyses. OS was stratified by timing of WGS (before versus after first systemic treatment), diagnostic category (known versus unknown primary) and whether biomarker-informed treatment was given following WGS. Median survival estimates were reported. HRs and 95% CIs were calculated using Cox proportional hazards regression. No prespecified hypothesis testing or multivariate adjustment for confounders was performed, in line with the exploratory and retrospective nature of this analysis.

### Reporting summary

Further information on research design is available in the Nature Portfolio Reporting Summary linked to this article.

## Online content

Any methods, additional references, Nature Portfolio reporting summaries, source data, extended data, supplementary information, acknowledgements, peer review information; details of author contributions and competing interests; and statements of data and code availability are available at 10.1038/s41591-026-04280-2.

## Supplementary information

Supplementary Information Supplementary Figs. 1 and 2.

Reporting Summary

Supplementary Tables 1–7 Supplementary tables. The first sheet of the table contains a list of all content within the different sheets of the file.

Supplementary Data 1 Supplementary data relating to the actionability grid as presented in Supplementary Fig. 1.

Supplementary Data 2 Supplementary data relating to the additional stratifications of OS analyses as presented in Supplementary Fig. 2.

## Source data

Source Data Figs. 1–5 and Extended Data Figs. 1, 3 and 5 Source Data Fig. 1: Summarized sample flow data for feasibility and simulated NGS-panel coverage. Source Data Fig. 2: Four sheets containing patient-level source data for each of the panels of the figure. Sheets Fig2A and Fig2B contain biomarker counts and diagnosis categories. Sheet Fig2C contains TMB figures and diagnosis categories. Sheet Fig2D contains tumor types and counts of biomarkers indicating reimbursed and experimental trial therapies. Source Data Fig. 3: Patient-level CUP solution outcomes and delivery of WGS-informed treatment. Source Data Fig. 4: Patient-level counts of parameters of clinically relevant results by diagnosis category. Source Data Fig. 5: Patient-level OS data with information for stratification by pretreatment status. Source Data Extended Data Fig. 1: Summarized turnaround time data. Source Data Extended Data Fig. 3: Summarized tumor-type distributions of the real-world study cohort and the Hartwig Medical database control cohort represented in the ‘cancer vignette’ visualizations of Extended Data Figs. 2 and 4. Source Data Extended Data Fig. 5: Patient-level details of genes and zygosity of detected PGVs; patients without detected variants are left out of the sheet.

## Data Availability

The curated clinical and genomic dataset required to reproduce the analyses and figures reported in this study is provided in Supplementary Tables 1–7 and Source Data provided with this paper and includes variables derived from routine clinical interpretation by an MTB. Patient-level raw clinical data are not publicly available due to privacy and governance restrictions but may be shared under controlled access in accordance with patient consent and applicable General Data Protection Regulation requirements. Data access for academic use may be requested via the Institutional Review Board of the Netherlands Cancer Institute (irb@nki.nl). Access is subject to IRB approval and requires a data transfer agreement with the Netherlands Cancer Institute. The estimated time to initial response is 4–6 weeks, and the expected total turnaround time is 4–6 months, including drafting and approval of a data transfer agreement. Raw and processed WGS data generated by Hartwig Medical Foundation for the study cohort are available for academic research under controlled access through Hartwig Medical Foundation’s data access request procedures, subject to data access board review and a data transfer agreement. The Hartwig Medical Database identifiers for the study cohort, which can be used to request access to additional genomic data, are provided in the Supplementary Tables. Requests can be submitted to dataaccess@hartwigmedicalfoundation.nl. Detailed request procedures, guidelines and forms can be found at https://www.hartwigmedicalfoundation.nl/en/data/data-access-request/. Source data are provided with this paper.

## Code availability

Custom code used for data analysis and figure generation is available at https://github.com/hartwigmedical/wgs-clinical-utility-solid-cancers/. Code used to generate the cancer vignettes (Extended Data Figs. 2 and 4) is available at https://github.com/Computational-Immunogenomics/vignettes-share/. The diagnostic WGS bioinformatics pipeline used by Hartwig Medical Foundation is open source and available at https://github.com/hartwigmedical/pipeline5
