# Author Correction: Real-world clinical utility of tumor whole-genome sequencing in solid cancers

**DOI:** 10.1038/s41591-026-04379-6

**Published:** 2026-04-02

**Authors:** Jeffrey van Putten, Petur Snaebjornsson, Linda J. W. Bosch, Roelof Koster, Paul Roepman, Joseph Usset, Mirjam C. Boelens, Tom van Wezel, Efraim H. Rosenberg, Serena Marchetti, Marieke Vollebergh, Doenja M. J. Lambregts, Lizet E. van der Kolk, Edwin Cuppen, Hilde H. Nienhuis, Kim Monkhorst

**Affiliations:** 1 Hartwig Medical Foundation, Amsterdam, The Netherlands; 2 Department of Pathology, Netherlands Cancer Institute, Amsterdam, The Netherlands; 3 Faculty of Medicine, University of Iceland, Reykjavik, Iceland; 4 Department of Pathology, Leiden University Medical Center, Leiden, The Netherlands; 5 Department of Medical Oncology, Netherlands Cancer Institute, Amsterdam, The Netherlands; 6 Department of Radiology, Netherlands Cancer Institute, Amsterdam, The Netherlands; 7 Department of Clinical Genetics, Netherlands Cancer Institute, Amsterdam, The Netherlands; 8 Center for Molecular Medicine, University Medical Center Utrecht, Utrecht, The Netherlands; 9 Department of Medical Oncology, University Medical Center Utrecht, Utrecht, The Netherlands

**Keywords:** Cancer genomics, Cancer of unknown primary

Correction to: *Nature Medicine* 10.1038/s41591-026-04280-2, published online 20 March 2026.

In the version of this article initially published, due to a version error, values listed on the right-hand side of Fig. 1 were incorrect, where in the text now reading “No actionable biomarkers detected: 196,” “Actionability detected only by simulated 523-gene panel and WGS: 153” and “Actionability detected by simulated 50-/523-gene panels and WGS: 352,” the values initially read “203,” “142,” and “344,” respectively. The figure is now amended in the HTML and PDF versions of the article.

