# Supplementary material for: Real-world clinical utility of tumor whole-genome sequencing in solid cancers
Source: Nat Med. 2026 Mar 20;32(4):1286–95. doi: 10.1038/s41591-026-04280-2 (PMC13099640; doi:10.1038/s41591-026-04280-2)

nature medicine

Supplementary information https://doi.org/10.1038/s41591-026-04280-2

Real-world clinical utility of tumor whole-genome sequencing in solid cancers

In the format provided by the authors and unedited

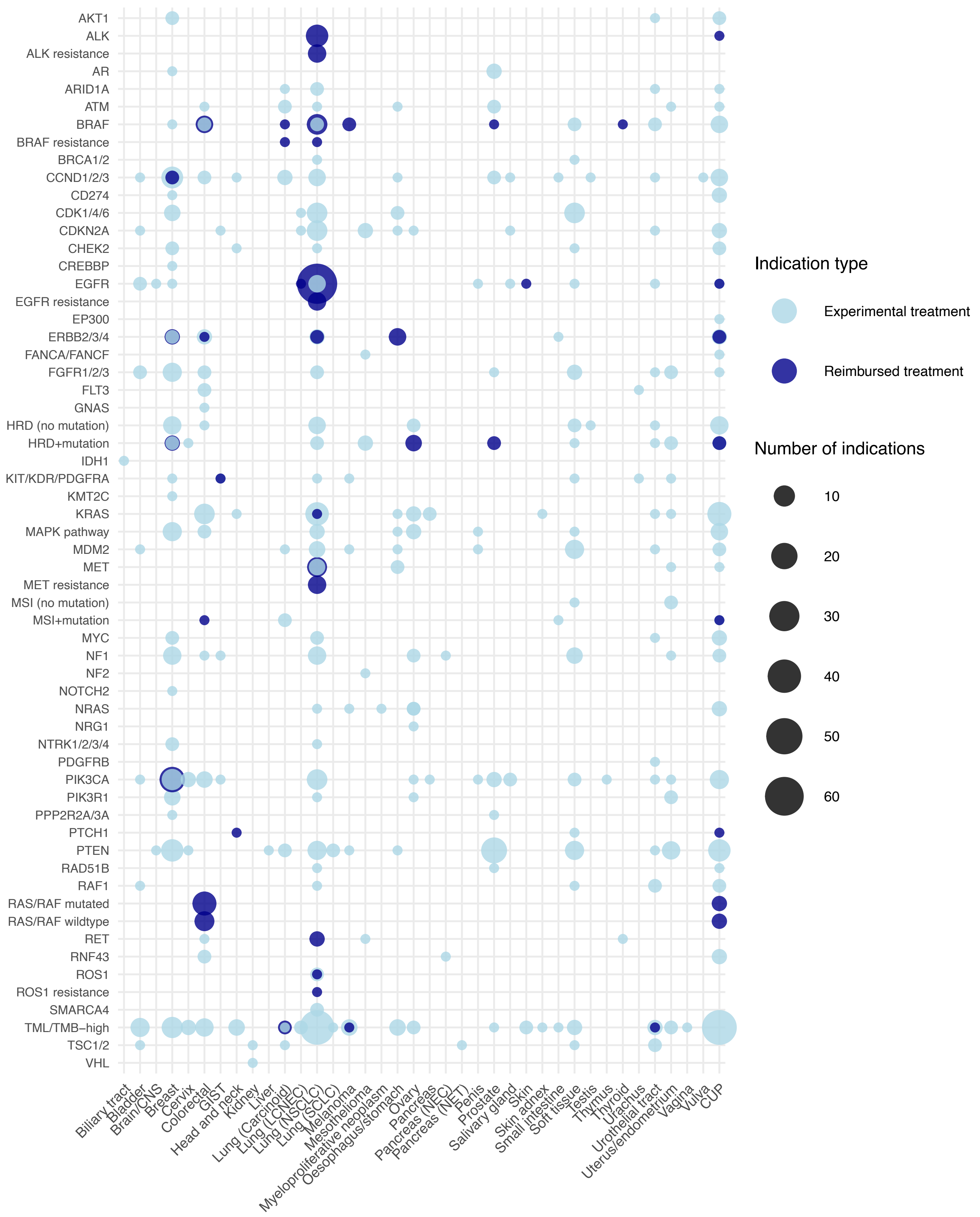
AKT1
ALK
ALK resistance
AR
ARID1A
ATM
BRAF
BRAF resistance
BRCA1/2
CCND1/2/3
CD274
CDK1/4/6
CDKN2A
CHEK2
CREBBP
EGFR
EGFR resistance
EP300
ERBB2/3/4
FANCA/FANCF
FGFR1/2/3
FLT3
GNAS
HRD (no mutation)
HRD+mutation
IDH1
KIT/KDR/PDGFRA
KMT2C
KRAS
MAPK pathway
MDM2
MET
MET resistance
MSI (no mutation)
MSI+mutation
MYC
NF1
NF2
NOTCH2
NRAS
NRG1
NTRK1/2/3/4
PDGFRB
PIK3CA
PIK3R1
PPP2R2A/3A
PTCH1
PTEN
RAD51B
RAF1
RAS/RAF mutated
RAS/RAF wildtype
RET
RNF43
ROS1
ROS1 resistance
SMARCA4
TML/TMB-high
TSC1/2
VHL
Biliary tract
Bladder
Brain/CNS
Breast
Cervix
Colorectal
GIST
Head and neck
Kidney
Liver
Lung (Carcinoid)
Lung (LCNEC)
Lung (NSCLC)
Lung (SCLC)
Melanoma
Mesothelioma
Myeloproliferative neoplasm
Oesophagus/stomach
Ovary
Pancreas
Pancreas (NEC)
Pancreas (NET)
Penis
Prostate
Salivary gland
Skin
Skin adnex
Small intestine
Soft tissue
Testis
Thymus
Thyroid
Urachus
Urothelial tract
Uterus/endometrium
Vagina
Vulva
CUP
Indication type
Experimental treatment
Reimbursed treatment
Number of indications
10
20
30
40
50
60

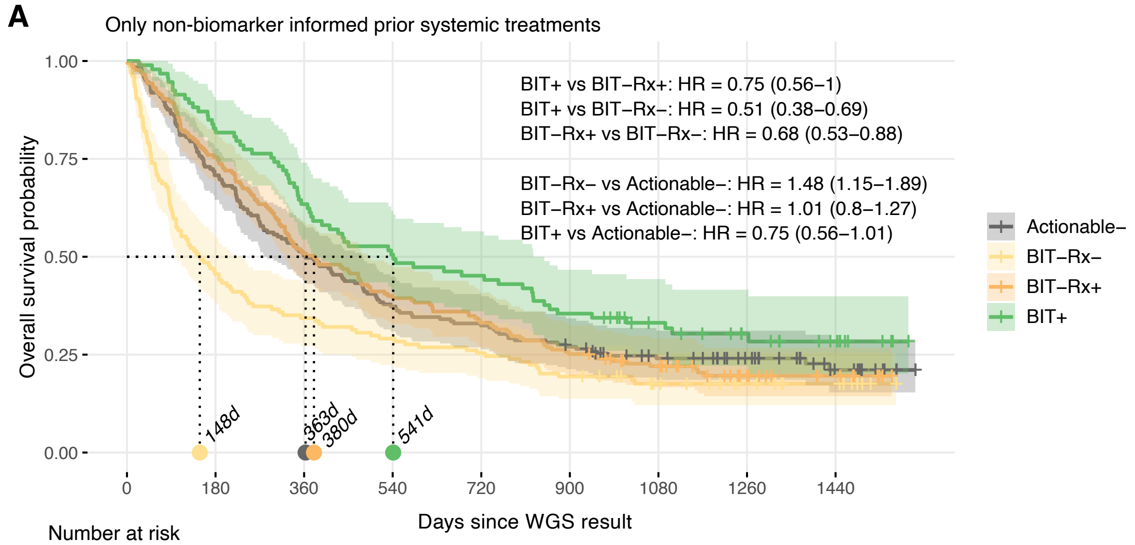

Number at risk

Actionable−	185	131	93	69	60	49	36	26	11
BIT−Rx−	134	61	46	39	34	26	17	11	7
BIT−Rx+	175	132	89	70	58	44	34	15	8
BIT+	93	77	59	47	42	33	24	15	9

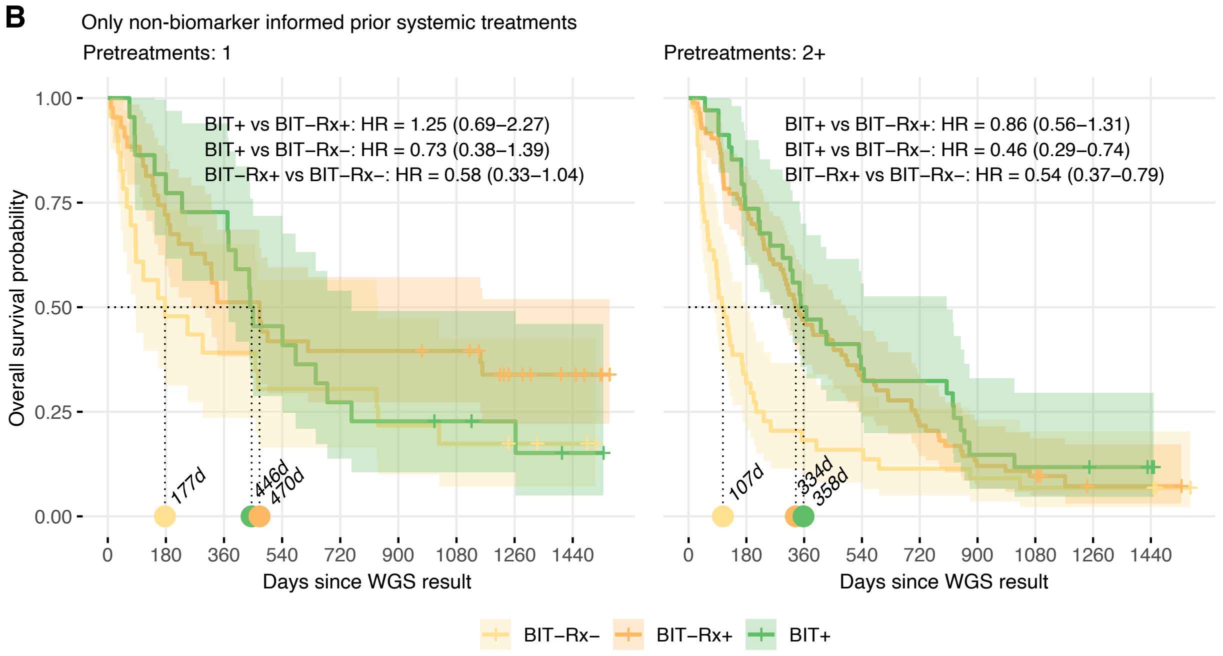

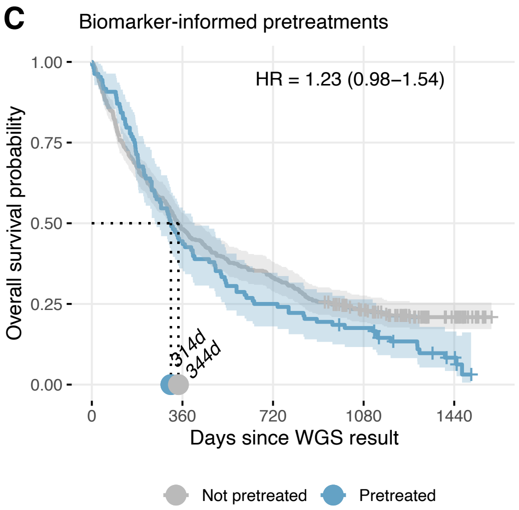

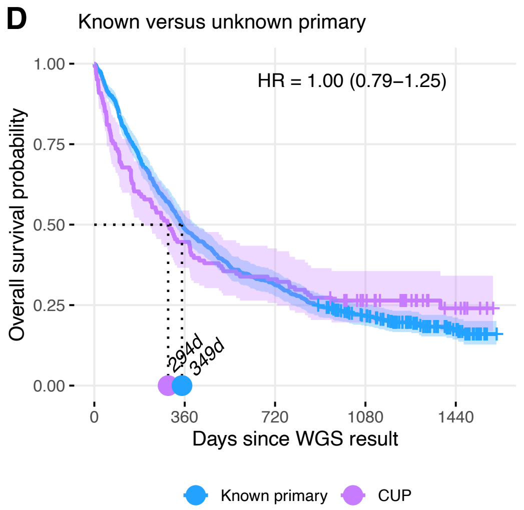

Supplement: Supplementary file 1 — Supplementary Figs. 1 and 2. [file 41591_2026_4280_MOESM1_ESM.pdf]
