# Supplementary material for: Real-world clinical utility of tumor whole-genome sequencing in solid cancers
Source: Nat Med. 2026 Mar 20;32(4):1286–95. doi: 10.1038/s41591-026-04280-2 (PMC13099640; doi:10.1038/s41591-026-04280-2)
Supplement: Supplementary file 2 — Reporting Summary [file 41591_2026_4280_MOESM2_ESM.pdf]

nature portfolio

Corresponding author(s): Edwin Cuppen, Kim Monkhorst

Last updated by author(s): Dec 18, 2025

Reporting Summary

Nature Portfolio wishes to improve the reproducibility of the work that we publish. This form provides structure for consistency and transparency in reporting. For further information on Nature Portfolio policies, see our Editorial Policies and the Editorial Policy Checklist.

Statistics

For all statistical analyses, confirm that the following items are present in the figure legend, table legend, main text, or Methods section.

n/a	Confirmed	
☐	☒	The exact sample size (*n*) for each experimental group/condition, given as a discrete number and unit of measurement
☐	☒	A statement on whether measurements were taken from distinct samples or whether the same sample was measured repeatedly
☐	☒	The statistical test(s) used AND whether they are one- or two-sided<br>*Only common tests should be described solely by name; describe more complex techniques in the Methods section.*
☐	☒	A description of all covariates tested
☐	☒	A description of any assumptions or corrections, such as tests of normality and adjustment for multiple comparisons
☐	☒	A full description of the statistical parameters including central tendency (e.g. means) or other basic estimates (e.g. regression coefficient) AND variation (e.g. standard deviation) or associated estimates of uncertainty (e.g. confidence intervals)
☒	☐	For null hypothesis testing, the test statistic (e.g. *F*, *t*, *r*) with confidence intervals, effect sizes, degrees of freedom and *P* value noted<br>*Give P values as exact values whenever suitable.*
☒	☐	For Bayesian analysis, information on the choice of priors and Markov chain Monte Carlo settings
☒	☐	For hierarchical and complex designs, identification of the appropriate level for tests and full reporting of outcomes
☒	☐	Estimates of effect sizes (e.g. Cohen's *d*, Pearson's *r*), indicating how they were calculated

Our web collection on statistics for biologists contains articles on many of the points above.

Software and code

Policy information about availability of computer code

Data collection	Clinical and genomic data and pathology information were extracted from HiX (ChipSoft, Amsterdam, the Netherlands) and LMS 5 (Telekom Healthcare Solutions, the Netherlands). Turnaround time data were derived from the external sequencing facility (Hartwig Medical Foundation, Amsterdam, the Netherlands).
Data analysis	Diagnostic genomic outputs were generated by Hartwig Medical Foundation using their open source, in-house WGS bioinformatics pipeline, available at https://github.com/hartwigmedical/<br>Data structuring, statistical analyses and visualisations for this study were performed in RStudio (version 2024.09.1+394; used R packages: networkD3 (v0.4), htmlwidgets (v1.6.4), extrafont (v0.20), readxl (v1.4.5), ggplot2 (v3.5.2), tidyr (v1.3.1), dplyr (v1.1.4), readr (v2.1.5), scales (v1.3.0), stringr (v1.5.1), cowplot (v1.1.3), survival (v.3.7-0), survminer (v.0.5.0) and patchwork (v1.3.0)).<br>Custom code used for data analysis and figure generation is available at https://github.com/hartwigmedical/wgs-clinical-utility-solid-cancers/.<br>Vignette code (for Extended Data Fig. 2 and 4) is available at https://github.com/Computational-Immunogenomics/vignettes-share/.

For manuscripts utilizing custom algorithms or software that are central to the research but not yet described in published literature, software must be made available to editors and reviewers. We strongly encourage code deposition in a community repository (e.g. GitHub). See the Nature Portfolio guidelines for submitting code & software for further information.

Data

Policy information about availability of data

All manuscripts must include a data availability statement. This statement should provide the following information, where applicable:

- Accession codes, unique identifiers, or web links for publicly available datasets
- A description of any restrictions on data availability
- For clinical datasets or third party data, please ensure that the statement adheres to our policy

The curated clinical and genomic dataset required to reproduce the analyses and figures reported in this study is provided in the Supplementary Tables and Source Data files, and includes variables derived from routine clinical interpretation by a molecular tumour board.
Patient-level raw clinical data are not publicly available due to privacy and governance restrictions, but may be shared under controlled access in accordance with patient consent and applicable GDPR requirements. Data access for academic use may be requested via the Institutional Review Board of the Netherlands Cancer Institute (irb@nki.nl). Access is subject to IRB approval and requires a data transfer agreement with the Netherlands Cancer Institute. The estimated time to initial response is 4-6 weeks, and the expected total turnaround time is 4-6 months, including drafting and approval of a data transfer agreement.
Raw and processed WGS data generated by Hartwig Medical Foundation for the study cohort are available for academic research under controlled access through Hartwig Medical Foundation's data access request procedures, subject to data access board review and a data transfer agreement. The Hartwig Medical Database identifiers for the study cohort, which can be used to request access to additional genomic data, are provided in the Supplementary Tables. Requests can be submitted to dataaccess@hartwigmedicalfoundation.nl. Detailed request procedures, guidelines and forms can be found at https://www.hartwigmedicalfoundation.nl/en/data/data-access-request/.

Research involving human participants, their data, or biological material

Policy information about studies with human participants or human data. See also policy information about sex, gender (identity/presentation), and sexual orientation and race, ethnicity and racism.

Reporting on sex and gender	Patients of male and female biological sex were included in this study. Patient-level data is available in Supplementary Table 1A, and aggregated data in Supplementary Table 1B.
Reporting on race, ethnicity, or other socially relevant groupings	No race, ethnicity, or other socially relevant groupings of interest were included to classify patients for the analyses.
Population characteristics	The population consists of all patients with at least one clinical whole genome sequencing request (n=1,052) at the Netherlands Cancer Institute's Antoni van Leeuwenhoek hospital, from January 2021 until the start of our analyses in November 2022. Detailed analyses of genomic and clinical data were performed in successfully sequenced patients with explicit consent (n=723). Only adults aged 18 or older are included, as no paediatric patients are treated at the Netherlands Cancer Institute.<br>Covariate-relevant characteristics recorded for the cohort included age at WGS reporting/request, biological sex, tumour type, and treatment categories where available (see Supplementary Tables).
Recruitment	Only clinically obtained data was used for this study. The decision to perform whole genome sequencing was based on clinicians' indication and/or on suggestion of a pathologist, without specific recruitment criteria other than an indication for broad molecular diagnostics.
Ethics oversight	The study was approved by the Institutional Review Board of the Netherlands Cancer Institute (IRBd22-294).

Note that full information on the approval of the study protocol must also be provided in the manuscript.

Field-specific reporting

Please select the one below that is the best fit for your research. If you are not sure, read the appropriate sections before making your selection.

☒ Life sciences ☐ Behavioural & social sciences ☐ Ecological, evolutionary & environmental sciences

For a reference copy of the document with all sections, see nature.com/documents/nr-reporting-summary-flat.pdf

Life sciences study design

All studies must disclose on these points even when the disclosure is negative.

Sample size	No statistical methods were used to calculate required samples size, as this was a retrospective cohort study. Sample size was determined by the number of eligible patients with a diagnostic WGS request in the hospital system.
Data exclusions	Before establishing the primary dataset, we excluded one patient for whom whole genomic sequencing was requested in a postmortem setting to determine hereditary predisposition as the aim of this request was of an essentially different nature compared to other clinical diagnostic requests. 63 patients who had a total 60 detailed whole genome sequencing reports did not have broad consent available and were therefore excluded from genomic and clinical analyses. 31 patients who indicated they did not wish to be informed on possible hereditary predisposition were excluded from the analysis in Extended Data Fig. 6; in main Figure 5, these patients were included for all

	studied parameters of clinical relevance with the exception of pathogenic germline variants detected. Four patients had no valid survival data from either the electronic health record or the national Personal Records Database and twelve patients had passed away before the WGS result date; these patients were thus excluded from the survival analyses.
Replication	Analyses were performed using scripted, version-controlled code (GitHub) on a curated analysis dataset provided in the Supplementary Tables. Key results and figures were regenerated from the same input dataset to verify reproducibility; no independent external replication cohort was used.
Randomization	No randomisation of the studied cohort was performed.
Blinding	Blinding to group allocation was not applicable, as this was a retrospective observational analysis of routinely collected clinical and genomic data; there was no experimental intervention or prospective assignment.

Reporting for specific materials, systems and methods

We require information from authors about some types of materials, experimental systems and methods used in many studies. Here, indicate whether each material, system or method listed is relevant to your study. If you are not sure if a list item applies to your research, read the appropriate section before selecting a response.

Materials & experimental systems

n/a	Involved in the study	
☒	☐	Antibodies
☒	☐	Eukaryotic cell lines
☒	☐	Palaeontology and archaeology
☒	☐	Animals and other organisms
☐	☒	Clinical data
☒	☐	Dual use research of concern
☒	☐	Plants

Methods

n/a	Involved in the study	
☒	☐	ChIP-seq
☒	☐	Flow cytometry
☒	☐	MRI-based neuroimaging

Clinical data

Policy information about clinical studies

All manuscripts should comply with the ICMJE guidelines for publication of clinical research and a completed CONSORT checklist must be included with all submissions.

Clinical trial registration	The study does not have a clinical trial registration as it does not concern an interventional trial. Approval for analyses of clinical and genomic data was received from the institutional review board of the Netherlands Cancer Institute Antoni van Leeuwenhoek hospital (IRBd22-294).
Study protocol	The methods used for data extraction and analyses are detailed in the Materials and Methods section.
Data collection	Genomic data from whole genome sequencing was generated from January 2021 until November 2022. Clinical data was obtained from the electronic health record and laboratory information management system between November 2022 and August 2023. Survival data was retrieved from the electronic health record and the national Personal Records Database in July 2025.
Outcomes	Overall clinical relevance was determined as main outcome, consisting of three parameters: (1) possibility for biomarker-informed reimbursed treatments based on molecular tumour board interpretations of genomic reporting as well as clinician's actions during the considered follow-up time, (2) diagnostic solution of Cancers of Unknown Primary or diagnostic improvement in previously established diagnoses, and (3) detection of clinically relevant pathogenic germline variants. Secondary outcomes follow items reported in the whole genome sequencing implementation study (study protocol: https://bmcmedgenomics.biomedcentral.com/articles/10.1186/s12920-020-00814-w; peer-reviewed article: https://doi.org/10.1002/path.5988).<br>Simulated NGS-panel coverage and overall survival of patients following WGS were reported as secondary outcomes.<br>Overall survival was analysed using Kaplan-Meier curves with stratification by tumour type, diagnosis category, number of prior treatment lines, and biomarker-informed pretreatment status. Effect sizes were reported as hazard ratios with 95% confidence intervals from Cox proportional-hazards models. Analyses were descriptive and reported as unadjusted estimates; no multivariable covariate adjustment was performed.

Plants

Seed stocks	*Report on the source of all seed stocks or other plant material used. If applicable, state the seed stock centre and catalogue number. If plant specimens were collected from the field, describe the collection location, date and sampling procedures.*
Novel plant genotypes	*Describe the methods by which all novel plant genotypes were produced. This includes those generated by transgenic approaches, gene editing, chemical/radiation-based mutagenesis and hybridization. For transgenic lines, describe the transformation method, the number of independent lines analyzed and the generation upon which experiments were performed. For gene-edited lines, describe the editor used, the endogenous sequence targeted for editing, the targeting guide RNA sequence (if applicable) and how the editor was applied.*
Authentication	*Describe any authentication procedures for each seed stock used or novel genotype generated. Describe any experiments used to assess the effect of a mutation and, where applicable, how potential secondary effects (e.g. second site T-DNA insertions, mosiacism, off-target gene editing) were examined.*
